# Neonatal Lupus erythematosus Following Rheumatoid Arthritis: Case Report and Literature Review

**Published:** 2014-05-16

**Authors:** Raheleh Assari, Vahid Ziaee, Mohammad-Hassan Moradinejad, Arash Mirmohammadsadeghi

**Affiliations:** 1 Growth and Development Research Center; 2 Pediatric Rheumatology Research Group, Rheumatology Research Center; 3 Department of Pediatrics; 4 Children’s Medical Center, Pediatrics Center of Excellence; 5 Department of Ophthalmology, Tehran University of Medical Sciences, Tehran, Iran

**Keywords:** Neonatal Lupus, Rheumatoid Arthritis, Anti SSA/Ro, Anti SSB/La

## Abstract

***Background:*** Neonatal lupus erythematosus (NLE) is a transient autoimmune disease of developing fetus and neonate in mothers with systemic lupus erythematosus (SLE). In this report we introduce an infant with NLE whose mother had rheumatoid arthritis.

***Case Presentation:*** Our case was a 40 day old male infant with discoid-like and annular skin lesions over forehead and neck, irritability and low grade fever. There was a history of prematurity due to preeclampsia. There was no cytopenia or cardiac involvement but liver enzymes were more than 5-fold increased. FANA, Anti Ro and La were negative. The mother had a history of un-controlled rheumatoid arthritis for 12 years with deformity in metacarpal and PIP and ulnar deviation in hands. FANA=1/640 and anti-SSB/La was positive in the mother but there was no other clinical and paraclinical sign of SLE. Without any treatment and during months, the skin and mucosal lesions gradually disappeared without any scar and liver enzymes reached the normal level. After 6 months follow up, he was symptom free with normal growth and development.

***Conclusion:*** We recommend to check anti SSA/Ro and anti SSB/La antibodies in all pregnant women with connective tissue diseases to prevent life-threatening involvement of the infant.

## Introduction

Neonatal lupus erythematosus (NLE) is an acquired autoimmune disease of developing fetus and neonate that is caused by transplacental passage of autoantibodies^[^^1^^]^. One of the most important clinical manifestations of NLE is cardiologic problems, including conduction abnormalities or a life-threatening cardiomyo-pathy without any conduction disorder^[^^1^^]^. The other presentations of NLE are cutaneous manifestations, characterized by annular or elliptical lesions of the face, trunk, scalp, or extremities^[^^2^^]^.

 The risk of NLE in mothers with Sjøgren syndrome (SS) or undifferentiated connective tissue disorder may be higher than in mothers with systemic lupus erythematosus (SLE)^[^^3^^]^. However, mothers with special autoantibodies sometimes do not have any clinical manifestation^[^^4^^]^. 

 In this article, we report on a case of neonatal lupus, whose mother was under treatment of active rheumatoid arthritis without any manifestation of SLE, Sjøgren syndrome or other connective tissue disease. 

## Case Presentation

**Fig. 1:**
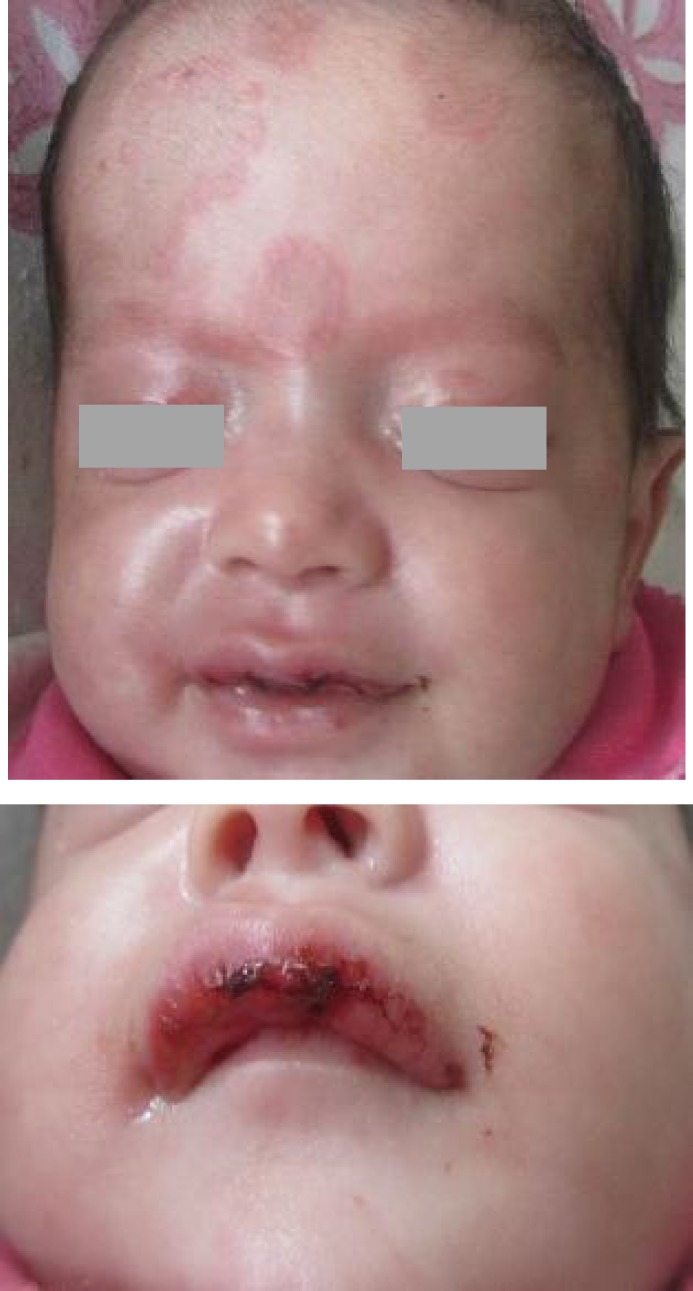
Annular skin lesions and mucosal lesions of the lips in an infant with neonatal lupus erythematosus.

A 40 days old male infant was referred to our clinic with discoid-like and annular skin lesions distributed over forehead and neck since one week ago. The parents complained of his irritability and low grade fever. Abnormal findings in physical examination were T=38.2°C, mild tachycardia (PR=140) and annular skin lesions on the face and mucosal lesions on the lips (Fig. 1). There was no cardiac arrhythmia or organo-megaly.

 In past history, the pregnancy was terminated at 35 weeks, due to preeclampsia. The neonate weighed 1300 gr at birth. According to the birth data and our examinations, his growth velocity was within normal limits.

 Abnormal laboratory studies were: Hgb 9.3, ESR 50, CRP 1, Serum glutamic pyruvic transaminase (SGPT) 310 IU/L (normal 25-45 IU/L), Serum glutamic oxaloacetic transaminase (SGOT) 285 IU/L (normal 37-43 IU/L). Other laboratory tests including platelets and neutrophil counts, serum creatinine, complement (C3) determination, urine analysis were normal. FANA and anti Ro (SSA) were negative in infant.

 Chext X-ray was normal and electrocardiogram showed tachycardia (140/min). To evaluate tachycardia, 24 hours holter monitoring and echocardiography were performed which had normal results.

**Fig. 2:**
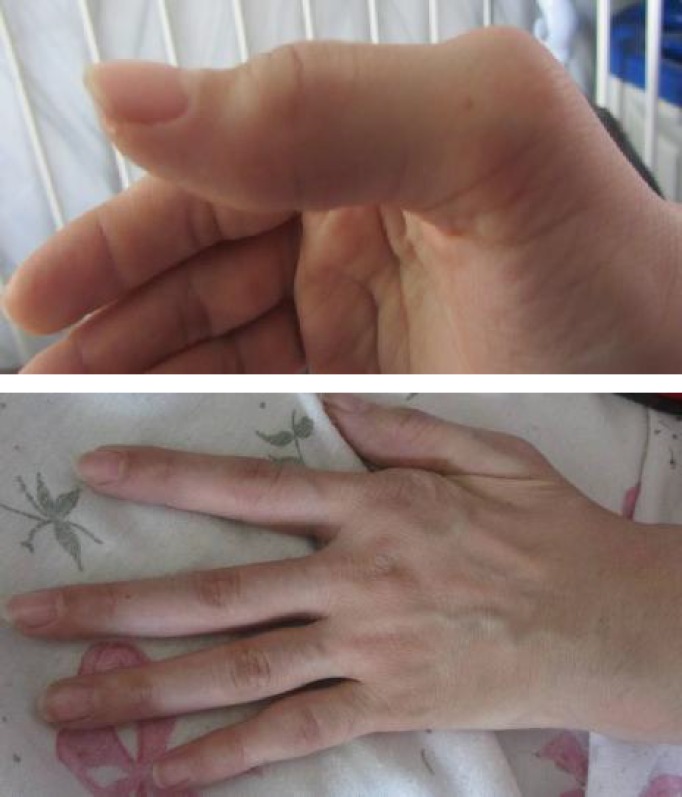
Arthritis in 2^nd^ Metacarpal and 1^st^ and 5^th^ PIP and ulnar deviation in the hand of the patient's mother.

 The mother was a known case of active rheumatoid arthritis for 12 years, under treatment during pregnancy with prednisolone and methyldopa. She had metacarpal and PIP deformities and ulnar deviation of the hands (Fig. 2). Her laboratory data during pregnancy showed FANA 1/640 but there was no clinical or laboratory findings compatible with SLE in 3 evaluations before and during pregnancy. Anti ds-DNA was negative and complements normal. Interestingly anti-SSB/La was positive (38 with normal range <20) during pregnancy but anti-Ro and anti-Sm antibodies were negative. 

After delivery, the laboratory tests for lupus were repeated. All the laboratory tests were normal, except for FANA=1/1280 and anti-SSB/La antibody which was higher than normal.

 The infant was followed without any treatment. In a period of 3 months, the skin lesions gradually disappeared without leaving any scars. The liver enzymes decreased and reached the normal level. 1 year follow up showed no new findings and normal growth and development of the infant.

## Discussion

Neonatal lupus (NL) is a disease of the fetus and neonate that results from transplacental passage of maternal anti-SSA/Ro and/or anti-SSB/La autoantibodies^[^^1^^]^. Transplacental passage of antibodies in NLE is associated with cardiac, skin and liver involvement^[^^5^^]^. Other less common manifestations are hematologic and neurologic problems^[^^5^^]^. The most significant site of involvement in NLE is the heart, causing conduction abnormalities, especially complete heart block (CHB)^[^^1^^]^. 

 Determination of true incidence of cutaneous neonatal lupus erythematosus (C-NLE) is difficult because the rash can be easily missed and resolve spontaneously^[^^6^^]^. These lesions often appear about 6 weeks after birth, and disappear after about 6 months when the autoantibodies are cleared from child`s circulation^[^^2^^]^. These lesions are similar to the lesions of subacute cutaneous lupus erythematosus (SCLE)^[^^7^^]^. The biopsy of these lesions shows basal cell damage with dermal mononuclear infiltration^[^^2^^]^.

 Hepatitis may be the only manifestation of NLE^[^^8^^]^. Liver disorders resolve spontaneously (like in our case), although death secondary to liver disorders was rarely reported before 6 months of age^[^^8^^]^.

 Most mothers of C-NLE infants are anti-SSA/Ro positive, although a few mothers were reported to have antibody to U1RNP without anti-SSA or anti-SSB^[^^9^^]^. Anti–52-kd Ro/SSA and anti-La/SSB antibodies play a more important role in cardiac manifestations than anti–60-kd Ro/SSA alone^[^^3^^]^. 

 These antibodies are necessary for pathogenesis of NLE but not sufficient. Maternal autoantibodies against RoRNP or other autoantigens such as U1RNP are also required for NLE pathogenesis^[^^9^^]^. Patients with SCLE have antibodies against proteins on RoRNP that are similar to antibodies in mothers of NLE infants^[^^7^^]^. However, many mothers with these antibodies do not deliver children with NLE. So, other factors are responsible in pathogenesis such as environ-mental triggers and genetics ^[^^10^^]^. 

 In the first reports, NLE was described as special skin and cardiac manifestations in the presence of maternal connective tissue disease, particularly SLE and SS. After that, NLE manifestations were seen in infants of mothers without any signs and symptoms of connective tissue disease^[^^11^^]^. After follow up for years, only a few number of these mothers developed manifestations of connective tissue disease, specially SS, SLE and pauci-undifferentiated autoimmune syndrome (P-UAS)^[^^4^^]^. The risk of acquiring such diseases is doubled in mothers with both anti-SSA/Ro and anti –SSB/La compared with mothers with anti-SSA alone^[^^4^^]^. As far as we know, our case is the first case with NLE manifestations whose mother was a known case of rheumatoid arthritis.

 Large prospective studies suggested that in mothers with anti-SSA/Ro, anti-SSB/La, or both, the risk of delivering NLE child is 1% to 2%^[^^12^^]^. Anti-SSB/La antibody had a higher specificity, since it was found only in the sera of mothers of children with NLE and not in sera from mothers of unaffected children, although it had a lower sensitivity of about 30%, because many mothers without anti-SSB /La had NLE infants^[^^13^^]^. Furthermore, Silverman and colleagues found positive anti-SSB/La in mothers with dermatologic NLE children and negative anti-SSB/La in mothers of CHB (complete heart block) children^[^^14^^]^. In our patient, despite negative anti-SSB/La, skin lesions were the main manifestations.

 On the other hand, 52KD anti-SSA/Ro antibodies were found in 85% of mothers of infants with CHB in some studies^[^^15^^]^. However, many mothers with SLE and anti-SSA/Ro antibody had an unaffected infant^[^^15^^]^. Recent studies demonstrated that antibody to p200 had greater correlation with CHB than 60KD or 52KD anti-SSA/Ro antibody^[^^15^^]^. Fortunately, our patient, regardless of positive anti SSA/Ro antibody, did not have any significant cardiac manifestation.

## Conclusion

This case of NLE had interesting findings such as rheumatoid arthritis with positive anti SSA/Ro antibody in the mother and no cardiac manifestation in infant. Our findings suggest that anti SSA/Ro and anti SSB/La antibodies must be checked in all pregnant women with connective tissue diseases to prevent dangerous cardiac complications in the infant.

## References

[1] Friedman DM, Kim MY, Copel JA (2008). Utility of cardiac monitoring in fetuses at risk for congenital heart block: the PR Interval and Dexamethasone Evaluation (PRIDE) prospective study. Circulation.

[2] Neinman AR, Lee LA, Weston WL (2000). Cutaneous manifestations of neonatal lupus without heart block: characteristics of mothers and children enrolled in a national registry. J Pediatr.

[3] Colombo G, Brucato A, Coluccio E (1999). DNA typing of maternal HLA in congenital complete heart block: comparison with systemic lupus erythematosus and primary Sjøgren’s syndrome. Arthritis Rheum.

[4] Rivera TL, Izmirly PM (2009). Disease progression in mothers of children enrolled in the Reseach Registry for Neonatal Lupus. Ann Rheum. Dis.

[5] Lee LA, Norris DA, Weston WL (1986). Neonatal lupus and the pathogenesis of cutaneous lupus. Pediatr Dermatol.

[6] Izmirly PM, Lianos C, Lee LA (2010). Cutaneous Manifestations of Neonatal Lupus and Risk of Subsequent Congenital Heart Block. Arthritis Rheum.

[7] Ng PP, Tay YK, Giam YC (2000). Neonatal lupus erythematosus: our local experience. Ann Acad Med Singapore.

[8] Cimaz R, Spence DL, Hornberger L, Silverman ED (2003). Incidence and spectrum of neonatal lupus erythematosus: a prospective study of infants born to mothers with anti-Ro autoantibodies. J Pediatr.

[9] Solomon BA, Laude TA, Shalita AR (1995). Neonatal lupus erythematosus: discordant disease expression of U1RNP-positive antibodies in fraternal twins-is this a subset of neonatal lupus erythematosus or a new distinct syndrome?. J Am Acad Dermatol.

[10] Smeenk RJ (1997). Immunological aspects of congenital atrioventricular block. Pacing Clin Electrophysiol.

[11] Press J, Uziel Y, Laxer RM (1996). Long-term outcome of mothers of children with complete congenital heart block. Am J Med.

[12] Ramsey-Goldman R, Hom D, Deng JS (1986). Anti-SS-A antibodies and fetal outcome in maternal systemic lupus erythematosus. Arthritis Rheum.

[13] Chan EK, Francoeur AM, Tan EM (1986). Epitopes, structural domains and asymmetry of amino acid residues in SS-B/La nuclear protein. J Immunol.

[14] Silverman ED, Buyon J, Laxer RM (1995). Autoantibody response to the Ro/La particle may predict outcome in neonatal lupus erythematosus. Clin Exp Immunol.

[15] Salomonsson S, Sonesson SE, Ottosson L (2005). Ro/SSA autoantibodies directly bind cardiomyocytes, disturb calcium homeostasis, and mediate congenital heart block. J Exp Med.

